# Poly ADP-Ribose Polymerase-1 inhibition by 3-aminobenzamide recuperates HEI-OC1 auditory hair cells from blast overpressure-induced cell death

**DOI:** 10.3389/fcell.2023.1047308

**Published:** 2023-03-06

**Authors:** Vijaya Prakash Krishnan Muthaiah, Kathiravan Kaliyappan, Supriya D. Mahajan

**Affiliations:** ^1^ Department of Rehabilitation Sciences, School of Public Health and Health Professions, University at Buffalo, Buffalo, NY, United States; ^2^ Department of Medicine, Division of Allergy, Immunology and Rheumatology, State University of New York at Buffalo, Clinical Translational Research Center, Buffalo, NY, United States

**Keywords:** *in vitro* blast injury model, PARP (poly(ADP-ribose) polymerase, 3-aminobenzamide, cell death, HEI-OC1 cells

## Abstract

**Introduction:** Poly ADP-Ribose Polymerase-1 (PARP1), a DNA repair enzyme is implicated as a key molecule in the pathogenesis of several neurodegenerative disorders. Traumatic insults inducing oxidative stress results in its over-activation causing inflammation and cell death (Parthanatos). As PARP1 inhibition is known to reduce oxidative stress, we hypothesized that PARP1 inhibition by a known inhibitor 3-aminobenzamide (3AB) might recuperate the damage in an *in vitro* model of blast injury using HEI-OC1 cells (mouse auditory hair cells).

**Methods:** Here, we evaluated the protective effect of 3AB on HEI-OC1 cells following single and repetitive blast overpressures (BOPs).

**Results:** We found that inhibition of PARP1 b 3AB inhibits the PARP1 enzyme and its action of a post-translational modification i.e. formation of Poly ADP-Ribose Polymers which leads to massive ATP depletion. PARP inhibition (3AB treatment) reduced the oxidative stress (4HNE, a marker of lipid peroxidation, and 8OHdG, a marker of oxidative DNA damage) in cells exposed to single/repetitive BOPS through up-regulation of Nrf2, a transcriptional regulator of antioxidant defense and the GCLC, a rate limiting enzyme in the synthesis of glutathione.

**Discussion:** Overall, we found that PARP inhibition by 3AB helps to maintain the viability of BOP-exposed auditory hair cells by recuperating the ATP pool from both mitochondrial and glycolytic sources.

## Introduction

In 2011, DOD Blast Injury Research Program Coordinating Office recommended prioritizing the identification of candidate pharmacologic strategies for early intervention to prevent damage to the cochlea and brain from blast injury. In 2020, VA’s annual report states that tinnitus and hearing loss contributed to 21.59% and 8.84% of the most prevalent disabilities respectively among veterans indicating that knowledge gaps still hold. Oxidative stress and inflammation are indicated as key components in the pre-clinical models of primary blast injury (rapid shifts in acoustic pressure) (Kocsis and Tessler, 2009) resulting in disruption of the blood-brain barrier (BBB) (Heyburn et al., 2019) with neuronal loss and microglial activation. Many antioxidants are being investigated as potential pharmacologic interventions for BOP injury, such as 2, 4-sulfonyl alpha phenyl tertiary butyl nitrone (HPN-07), and N-acetylcysteine (NAC). However, oxidative stress signaling pathways converges on the PARP-1 over-activation due to increased reactive oxygen and nitrogen species following traumatic insults. Evidence indicates inhibition of Poly ADP-Ribose Polymerase-1 reduces neuroinflammation and helps to maintain the integrity of BBB (Rom et al., 2016).

In post-traumatic insults, over-activation of a DNA repair enzyme, Poly ADP-Ribose Polymerase-1 (PARP-1), functions as an epicenter of cellular stress responses and disrupts cellular energetics through several signaling pathways (Luo and Kraus, 2012). Oxidative stress induced over-activation of PARP1 results in formation of Poly ADP-Ribose (PAR) polymers on itself and other nuclear proteins. PARP1 being a NAD^+^ dependent nuclear enzyme this process of PAR polymer formation leads to NAD^+^ depletion due to over-consumption (Alano et al., 2010). The turnover requires NAD^+^ synthesis by NMNAT (Nicotinamide mononuncleotide adenyltransferase-1) from NMN (Nicotinamide mononucleotide) which requires ATP (Luo and Kraus, 2012). Excess PARylation, a post-translational modification, by PARP1 over-activation consumes NAD^+^ (Nicotinamide adenine dinucleotide) and ATP to form PAR polymers comprising up to ∼200 ADP-ribose chains through a covalently attached ribose-ribose branched linkages on amino acid residues of several nuclear proteins (Krüger et al., 2020). Thus, the ATP and NAD^+^ depletion through PARP-1 over-activation (Fouquerel et al., 2014) occurs as a result of PARylation (Wei and Yu, 2016) leading to cell death, which is often referred to as Parthanatos (Liu et al., 2022). Hence, PARP-1 has been implicated as a key molecule in the pathomechanism of several neurodegenerative disorders and is considered a potential therapeutic target (Arruri et al., 2021; Thapa et al., 2021). Recently, four PARP inhibitors (PARPi) namely olaparib, rucaparib, niraparib, and talazoparib were currently FDA-approved for their clinical use in cancer chemotherapy (Sisay and Edessa, 2017; Wolford et al., 2017; Boussios et al., 2020). Increased evidence of PARPi in preventing post-traumatic neuronal loss (Stoica et al., 2014) and suppression of microglial activation (d’Avila et al., 2012) sets stage for investigation of beneficial effect of PARP inhibition on non-oncological diseases especially on the diseases with high severity and limited therapeutic options. Towards the repurposing of matured drugs, pre-clinical and clinical action plan are being developed (Berger et al., 2018).

As 3-Aminobenzamide (3AB) or other PARP inhibitors have been investigated for their therapeutic benefits in many neurodegenerative conditions by reversing mitochondrial dysfunction and ATP pool (Muthaiah et al., 2017), which potentially inhibits both oxidative stress and neuronal cell death, in the present study, we evaluated the effect of a known potent PARP-1 inhibitor, 3AB on mouse auditory hair cell line HEI-OC1 cells following single and repetitive blast overpressures.

## Materials and methods

### Cell culture and blast over-pressure (BOP)

**FIGURE 1:**
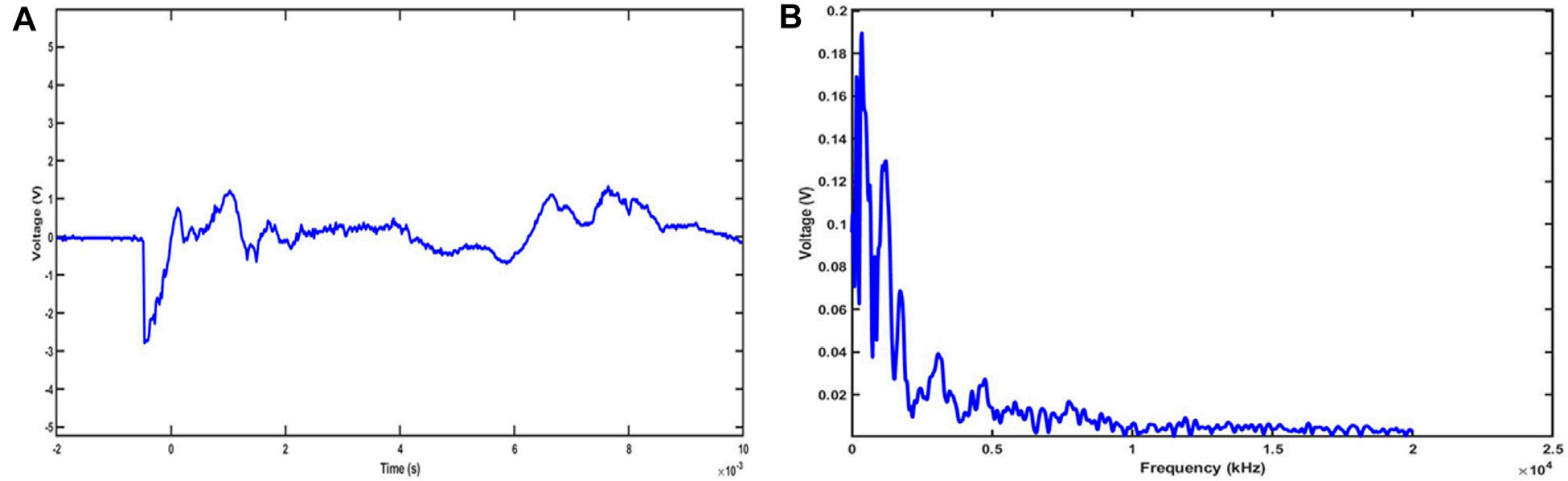
Representative Impulse waveform **(A)** and frequency spectrum **(B)** of blast overpressure at 23 psi (∼172 dB pSPL).

The House Ear Institute-Organ of Corti 1 (HEI-OC1) cells are widely used auditory cell lines that are derived from transgenic mouse auditory organs. The HEI-OC1 cells used for this study were kindly gifted by Professor Federico Kalinec (House Ear Institute, Los Angeles, CA, United State). The cells were grown and maintained under permissive conditions (33°C & 10% CO2) in high glucose DMEM (Corning, 10–017-CV) with 10% fetal bovine serum (Cat# F2442; Sigma Aldrich, United State) supplemented with 1% Amphotericin B (Cat# 120–097–711; Quality Biologicals, United State) and penicillin (10000 U/L). The cells were grown (5 × 10^5^) in 100 mm culture dishes and exposed to an impulse blast wave (∼170 dB SPL; <2 ms). The *in vitro* model of blast exposure was established using a custom acoustic shock tube (similar to the design of the National Institute for Occupational Safety and Health (NIOSH) and developed by Mark Cauble Precision Inc. A similar kind of shock tube was employed by Hickman et al., 2018 (Niwa et al., 2016), a study that used Chinchilla to study blast-induced cochlear synaptopathy (Kaliyappan et al., 2021; Schmitt et al., 2021; Zemaitis et al., 2021). The Impulse wave and frequency spectrum of the blast overpressure were represented in Figure 1. After BOP, the treated group’s cells were incubated in 3 mM 3-aminobenzamide (3AB) (Cat# 339080050; Acros Organics, United State) in culture media till the end of the experimental timeline. The study groups include sham-exposed, 1-BOP, 3-BOP, 1-BOP treated with 3AB (1-BOP/3AB), and 3-BOP treated with 3AB (3-BOP/3AB). The 1-BOP group cells receive a single blast exposure, whereas the 3-BOP group cells receive 3 blast exposures at an interval of 1 minute.

### Cell viability after blast overpressure

The cell viability and cell proliferation were determined by the sensitive colorimetric Cell Counting Kit-8 (CCK-8) assay (Cat# CK04-05; Doiindo, Rockville, United State) at post-BOP 24 h. In brief, a total of 1 × 10^4^ cells were seeded in each well of 96 well plates, cultured overnight, and exposed to BOP (∼170 dB SPL). The cell viability at post-BOP 24 h was determined by adding 10 μL of CCK-8 solution to each well of the plate. After 2 h of incubation at 37°C, the absorbance was measured at 450 nm using the microplate reader (BioTek, United State). The assay was repeated three times (*n* = 3).

### Detection of mitochondrial membrane potential depolarization

The mitochondrial membrane potential post-BOP was assessed by the Cell Meter™ JC-10 Assay Kit (AAT Bioquest, CA, United State). The cells were seeded (1 × 104/well) in 96 well plates, cultured for 12 h, and exposed to single BOP. After 24 h post-BOP, the treated cells were added with 50 μL/well of JC-10 dye-working solution (1 part of Component A+ 100 parts of Component B) and incubated at 37°C in dark for 60 min. Then, 50 μL of Component C was added to each well and the fluorescence intensities were measured on a microplate reader (BioTek, United State) using dual fluorescence (Ex/Em = 490/525 nm, Cutoff = 515 nm, and 540/590 nm, Cutoff = 570 nm) with bottom read mode. The ratio of green/red was calculated to denote the extent of mitochondrial depolarization.

### Cellular and mitochondrial ATP production

The total ATP production, non-mitochondrial cellular ATP production (glycoATP) through the glycolytic pathway, and mitochondrial ATP production (mitoATP) through oxidative phosphorylation (OXPHOS) were measured using XFp Real-Time ATP rate assay (Agilent Technologies, Santa Clara, United State). The sum of glycoATP and mitoATP provides the total cellular ATP production in the cells. The glycoATP was measured by measuring the Proton efflux rate (PER), whereas the mitoATP was determined by measuring the difference in oxygen consumption (Oxygen consumption rate, OCR) after the addition of mitochondrial inhibitors (oligomycin, inhibits Complex V and Rotenone/Antimycin mix inhibits Complex I/III). For ATP measurement, a total of 5 × 10^5^ cells were cultured in a 100 mm culture plate and exposed to BOP and the cells were subjected to real-time ATP rate assay at post-BOP 24 h by transferring them to a calibrated seahorse XF tissue-culture plate (5 × 10^4^ cells/well). The assay was repeated three times (*n* = 3).

### Quantification of mRNA expression of PARP1, NRF2 & GCLC

The mRNA expression levels of PARP1, NRF2, and GCLC following BOP were evaluated through a quantitative polymerase chain reaction (qPCR) procedure. The total RNA from the HEI-OC1 cells was extracted using TRIzol™ Reagent (Cat# 15596026; Thermo Fisher Scientific, United State) and the purity and concentration were determined in NanoDrop™ One (Thermo Scientific, United State). Using the High-Capacity cDNA Reverse Transcription Kit (Cat# 4368814; Thermo Fisher Scientific, United State), the cDNA synthesis was carried out. The qPCR was performed in CFX96 Touch Real-Time PCR Detection System (Bio-Rad, United State). The reaction mixture includes the cDNA template, forward and reverse primers (Integrated DNA Technologies Inc., United State), and 2X SsoAdvanced™ universal SYBR^®^ Green Supermix (Bio-Rad, United State). The reaction conditions were set as follows: Polymerase Activation and DNA Denaturation for 30 s at 95°C; Denaturation for 15 s at 95°C; Annealing/Extension for 30 s at 60°C; 40 cycles. Details of the mouse primers used in this study were listed in Table 1. The GAPDH was used as the internal control for the assessment. The relative gene expression was calculated by the relative quantification method (2^−ΔΔCT^) using CFX Manager software (Bio-Rad, United State). The amplified genes were confirmed by analyzing the melt curves. The experiment was carried out using sample triplicates (*n* = 3).

### Immunoblotting for protein assessments

The relative protein expression of PARP1, Poly (ADP-ribose) polymer (PAR), 4-Hydroxynonenal (4HNE), and 8-Oxo-2′-deoxyguanosine (8-OHDG) were assessed by the immunoblotting procedure. The cell lysate was prepared by adding RIPA lysis buffer (Cat# sc-24948; Santa Cruz Biotechnology, United State) and centrifugation. The total protein concentration of the samples was determined by the Pierce™ BCA Protein Assay kit (Cat# 23227; Thermo Fisher Scientific, United State). An equal amount of protein (20 µg) was separated by SDS-PAGE along with the protein Standards (Cat# 1610374; BioRad, United State) and transferred into PVDF Transfer Membrane (Cat# 88518; Thermo Fisher Scientific, United State) using the Wet transfer method (constant 100 v for 90 min). The blots were washed with washing buffer and blocked for 5 min using Everyblot blocking buffer (Cat# 12010020; BioRad, United State). Further, the blots were incubated with Anti-PARP (Cat# 9542; Cell Signaling Technology, United State), Anti-PAR (Cat# 83732; Cell Signaling Technology, United State), Anti-4HNE (Cat# ab46545; Abcam, United State), and Anti- 8-OHdG (Cat# BS-1278R; Bioss Antibodies Inc., United State) rabbit polyclonal antibodies (1:2000) for overnight at 4°C. Then, the blots were washed and incubated in pre-diluted (1:5000) HRP conjugated Anti-Rabbit secondary antibodies (Cat# 7074; Cell Signaling Technology, United State) at room temperature for 1 h and washed thrice with washing buffer. The blots were imaged in ChemiDoc™ MP Imaging System using SuperSignal™ West Femto Maximum Sensitivity Substrate (Cat# 34095; Thermo Fisher Scientific, United State) and analyzed using Image Lab™ Software (BioRad, United State). All the protein band intensities were normalized against internal control protein, βActin (Cat# sc-47778; Santa Cruz Biotechnology, United State), and sham samples.

### Statistical analysis

The statistical analysis was performed using GraphPad Prism V6.01 (GraphPad Software, La Jolla, CA). The results are expressed as mean ± standard error of the mean. The statistical significance was assessed by one-way analysis of variance with Tukey’s “*post hoc*” test. The results with *p* ≤ 0.05 were considered statistically significant.

## Results

### Effect of 3AB on post-BOP cell viability

**FIGURE 2:**
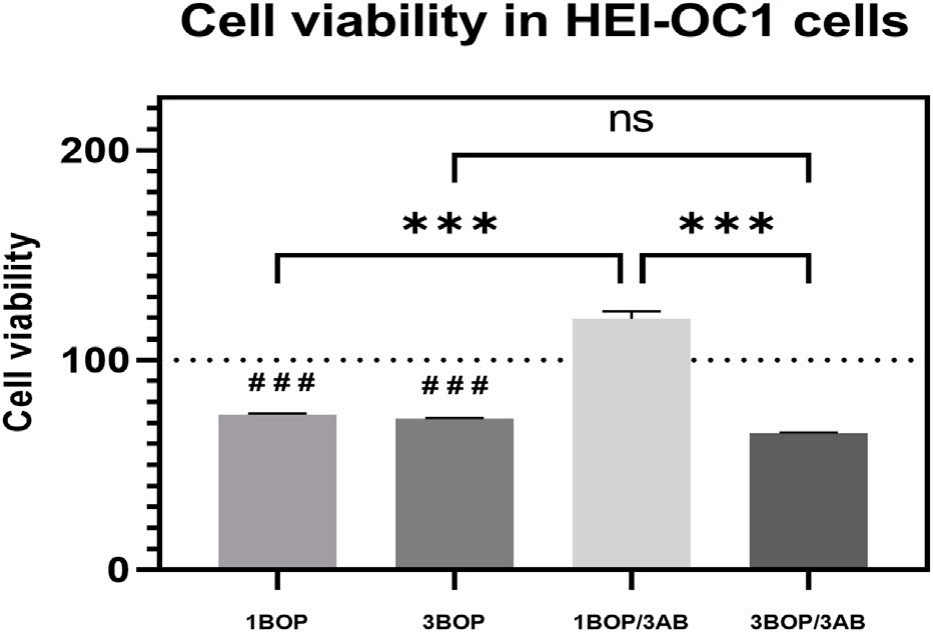
The histogram show the cell viability using CCK8 assay in HEI-OC1 cells following BOP/3AB treatment (3 mM). ***p < 0.001 among groups; ###*p* < 0.001 versus sham group. Values are represented as Mean ± SEM (*n* = 3).

The cell viability at 24 h after BOP was assessed using the CCK-8 assay. The results clearly showed (Figure 2) that the cell viability was significantly reduced in the 1-BOP, and 3-BOP group (###*p* < 0.001; F (4, 10) = 139.4) compared to sham cells. The cell viability in the 1-BOP/3AB group cells was significantly higher (***p < 0.001; F (4, 10) = 139.4) when compared to 1-BOP cells. But the 3-BOP/3AB cell viability was not improved when compared with 3-BOP cells. Additionally, there is no significant difference in the cell viability between 1-BOP and 3-BOP group cells (F (4, 10) = 139.4).

### Effect of 3AB on post-BOP mitochondrial membrane potential

**FIGURE 3:**
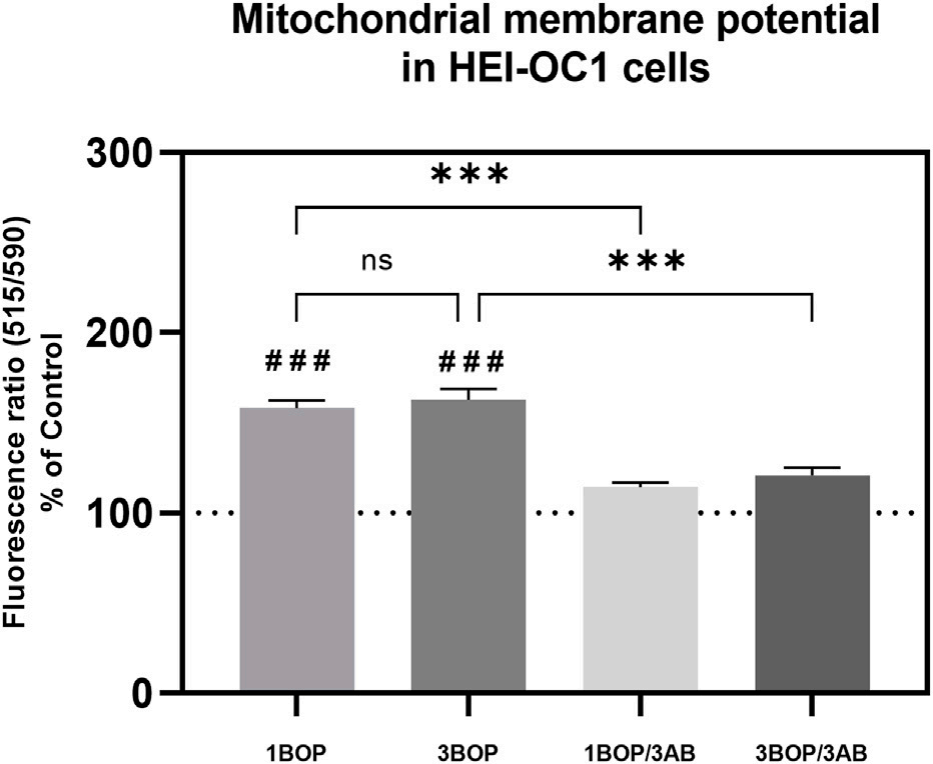
The histogram shows the mitochondrial membrane potentials using JC10 assay in HEI-OC1 cells following BOP/3AB treatment (3 mM). ***p < 0.001 among groups; ###*p* < 0.001 versus sham. Values are represented as Mean ± SEM (*n* = 3).

The mitochondria membrane potential changes in the HEI-OC1 cells after blast exposure and 3AB treatment were analyzed using the JC-10 Mitochondrial Membrane Potential Assay Kit. The result (Figure 3) displayed a significant increase (###*p* < 0.001; F (4, 10) = 48.25) of the mitochondrial membrane potential in the 1-BOP and 3-BOP cells when compared with the sham-exposed cells. This mitochondrial membrane potential was significantly reduced (***p < 0.001; F (4, 10) = 48.25) in 3AB-treated 1-BOP/3AB and 3-BOP/3AB group cells. There is no significant difference in mitochondrial membrane potential between the 1-BOP and 3-BOP group cells (F (4, 10) = 48.25).

### Effect of 3AB on post-BOP cellular energetics

**FIGURE 4:**
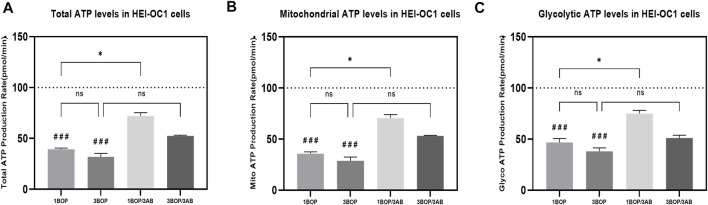
Effect of BOP on the cellular ATP levels of HEI-OC1 cells after blast overpressure using Seahorse XF 24 Analyzer. The histogram shows the **(A)** TotalATP, **(B)** mitoATP & **(C)** GlycoATP in HEI-OC1 cells following BOP/3AB treatment (3 mM). *p < 0.05; ###*p* < 0.001 versus sham. Values are represented as Mean ± SEM (*n* = 3).

The glycolytic pathway-mediated glycoATP production and oxidative phosphorylation-mediated mitoATP production were measured through Seahorse XFp Real-time ATP rate assay. The results (Figure 4) indicated a significant decrease (###*p* < 0.001) of glycoATP (F (4, 10) = 23.79), mitoATP (F (4, 10) = 22.17) and total ATP (F (4, 10) = 23.91) production in1-BOP and 3-BOP groups cells. Though the ATP production was lower in 3-BOP cells, it was significant against the 1-BOP group. The reduced glycoATP (F (4, 10) = 23.79), mitoATP (F (4, 10) = 22.17), and total ATP (F (4, 10) = 23.91) productions were significantly increased (*p < 0.05) in 1-BOP/3AB treatment group cells when compared with 1-BOP cells. However, the ATP production was not improved in 3-BOP/3AB group cells compared with 3-BOP cells.

### Effect of 3AB on post-BOP mRNA expression of PARP1, NRF2 & GCLC

**FIGURE 5:**
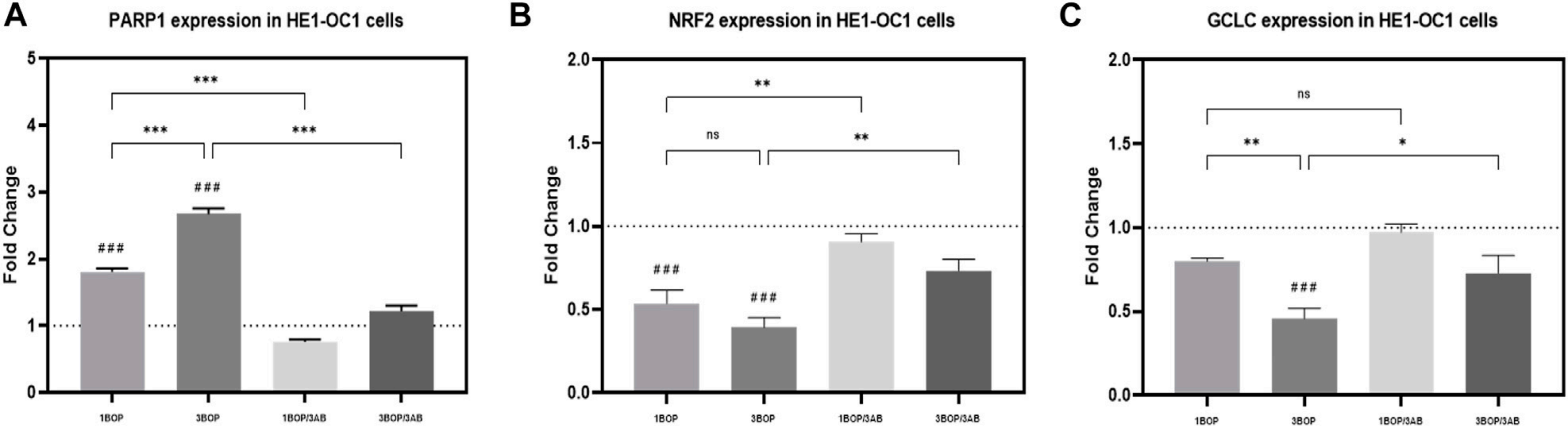
Reverse transcription-quantitative polymerase chain reaction: The histograms represents the mRNA expressions of **(A)** PARP1, **(B)** NRF2 & **(C)** GCLC in HEI-OC1 cells following BOP/3AB treatment (3 mM). *p < 0.05; **p < 0.005; ***p < 0.001 among groups; ###*p* < 0.001 versus sham. Values are represented as Mean ± SEM (*n* = 3).

The changes in PARP1, NRF2, and GCLC mRNA expression levels, post-BOP and 3AB treatment were assessed by qPCR in HEI-OC1 cells (Figure 5). The qPCR results indicated a significant increase in the PARP1 expression (###*p* < 0.001; F (4, 25) = 166.2), with a significant decrease in NRF2 expression (###*p* < 0.001; F (4, 25) = 18.84) in 1-BOP and 3-BOP cells when compared to sham exposed cells. Additionally, the PARP1 expression was significantly higher in the 3-BOP group against 1-BOP cells. However, the PARP1 expression was significantly decreased (***p < 0.001; F (4, 25) = 166.2), with a significantly increased NRF2 expression (**p < 0.005; F (4, 25) = 18.84) in 1-BOP/3AB and 3-BOP/3AB group cells when compared with 1-BOP and 3-BOP cells respectively. Further, the GCLC expression was only reduced significantly in 3-BOP cells (###*p* < 0.001; F (4, 25) = 13.67), not in the 1-BOP group when compared to the sham group. Though the 25) = 13.67).

### Effect of 3AB on post-BOP protein expression

**FIGURE 6:**
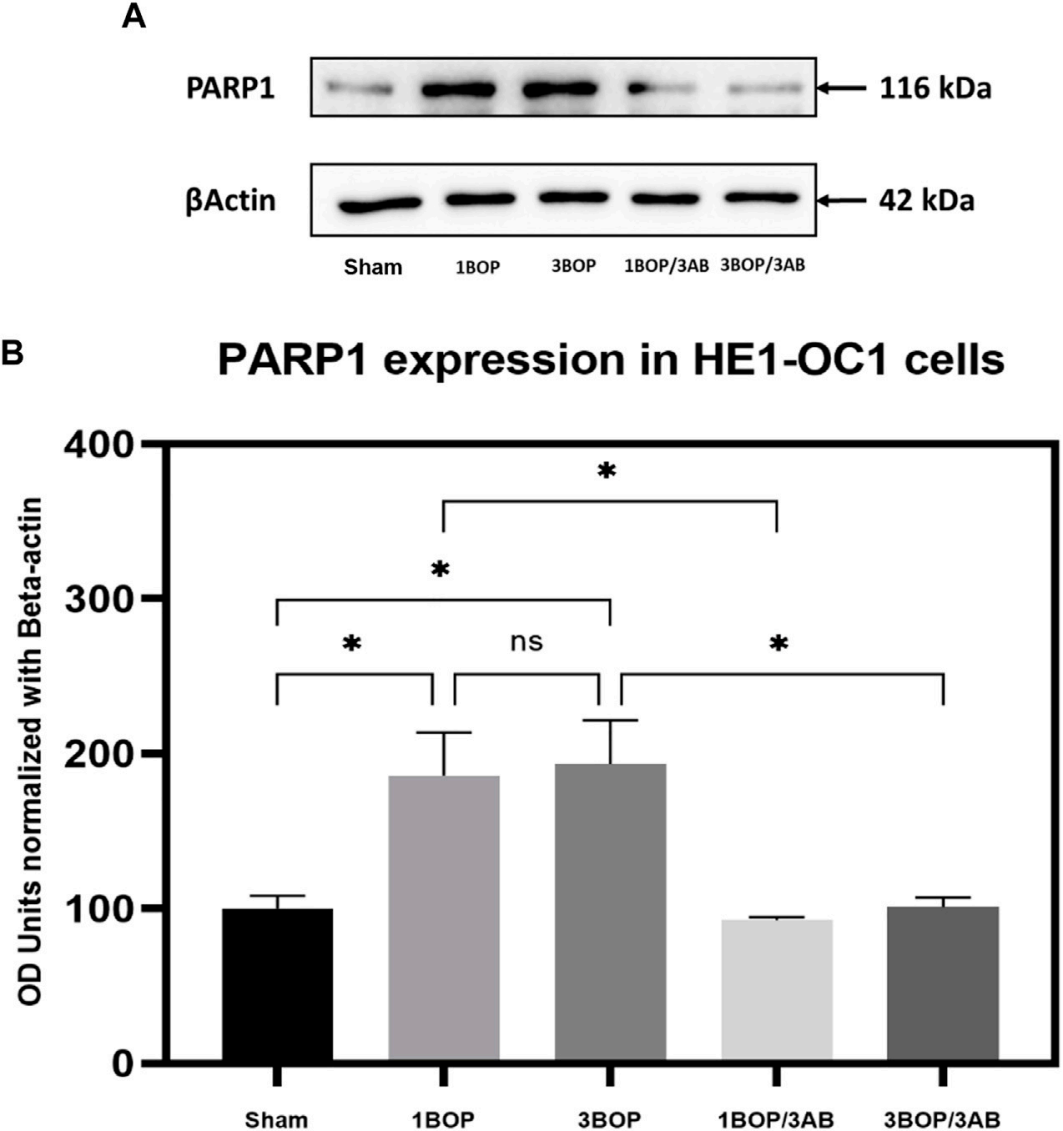
Assessment of PARP1 protein expressions by immunoblotting analysis in HEI-OC1 after BOP exposure. The **(A)** immunoblots shows the protein expressions of PARP1 levels in HEI-OC1 cells. **(B)** The histogram shows the semi-quantitative protein expression of PARP1 levels in HEI-OC1 cells normalized against the internal control β-Actin. Statistical difference between the cell groups were (*p < 0.05) represented as Mean ± SEM (*n* = 3).

**FIGURE 7:**
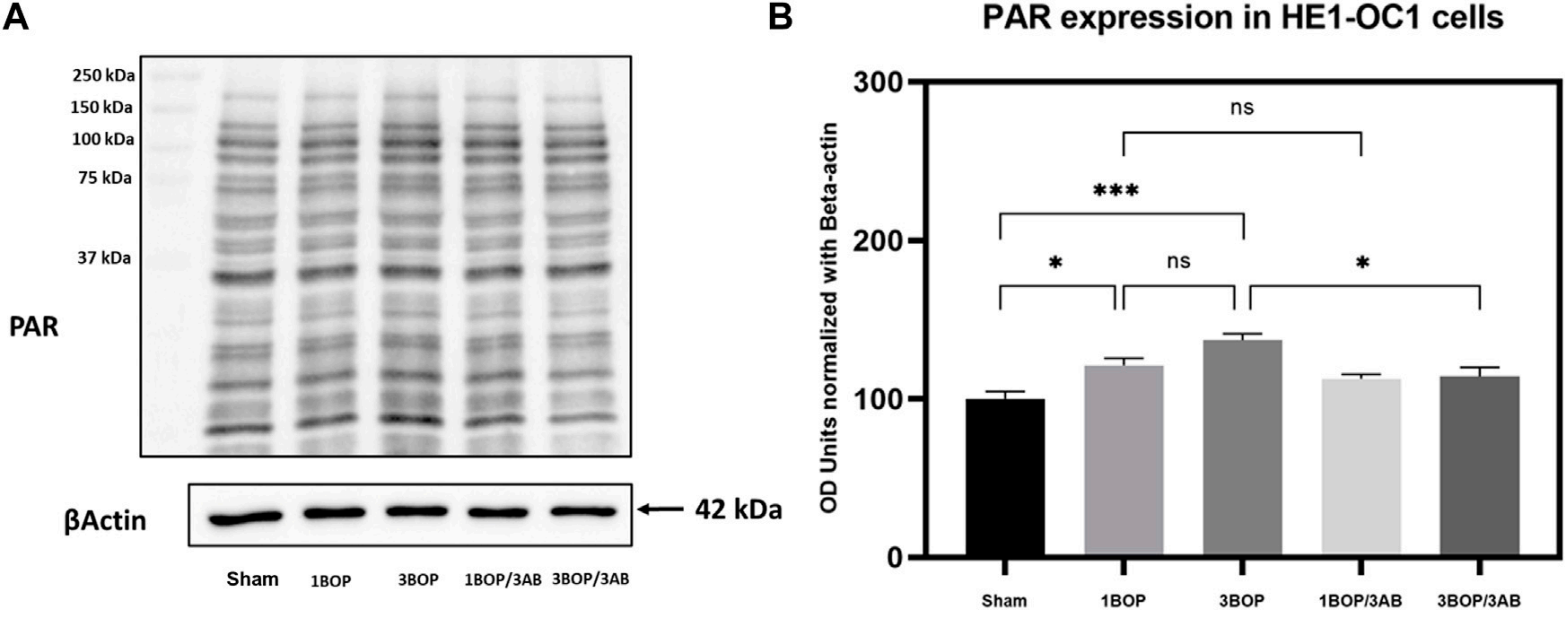
Assessment of PAR protein expressions by immunoblotting analysis in HEI-OC1 after BOP exposure. The **(A)** immunoblots shows the protein expressions of PAR levels in HEI-OC1 cells. **(B)** The histogram shl e semi-quantitative protein expression of PAR levels in HEI-OC1 cells normalized against the internal con ES-Actin. Statistical difference between the cell groups were (*p < 0.05; **p < 0.001) represented as Mean ± SEM (*n* = 3).

**FIGURE 8:**
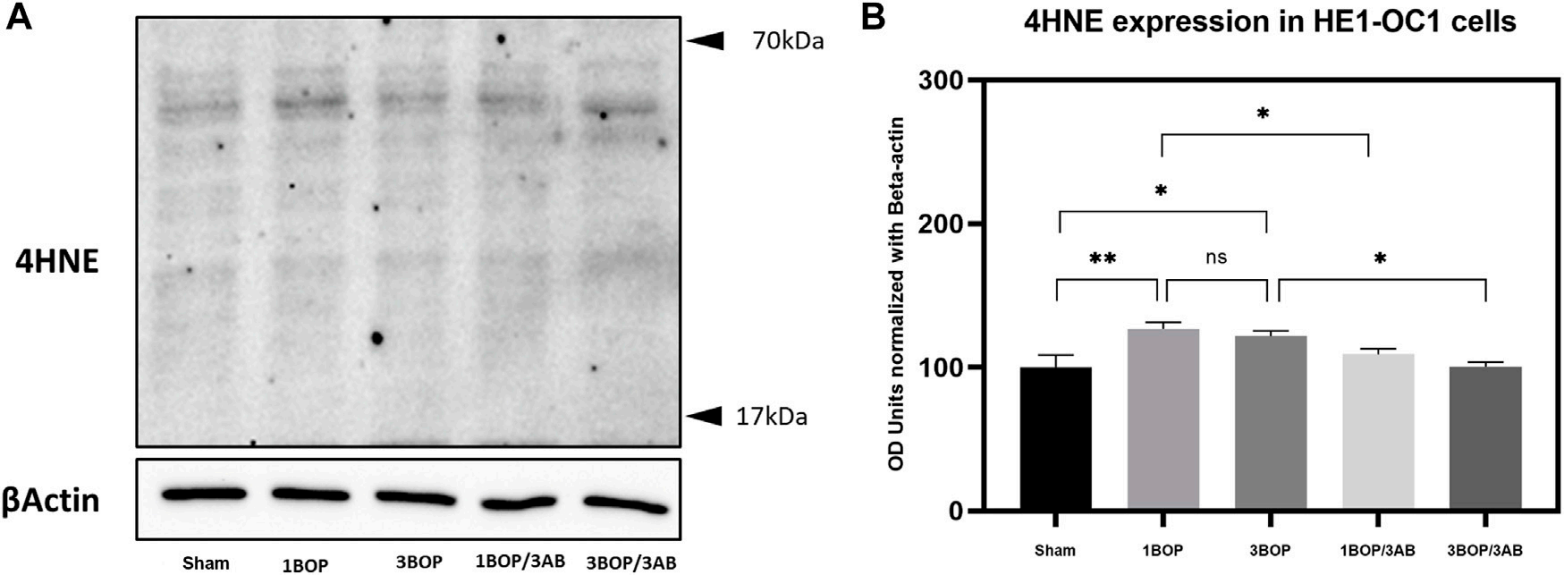
Assessment of 4HNE protein expressions by immunoblotting analysis in HEI-0C1 after BOP exposure. The **(A)** immunoblots shows the protein expressions of 4HNE levels in HEI-OC1 cells. **(B)** The histogram shows the semi-quantitative protein expression of 4HNE levels in HEI-OC1 cells normalized against the internal control 13-Actin. Statistical difference between the cell groups were (*p < 0.0S; **p < 0.005) represented as Mean ± SEM (*n* = 3).

**FIGURE 9:**
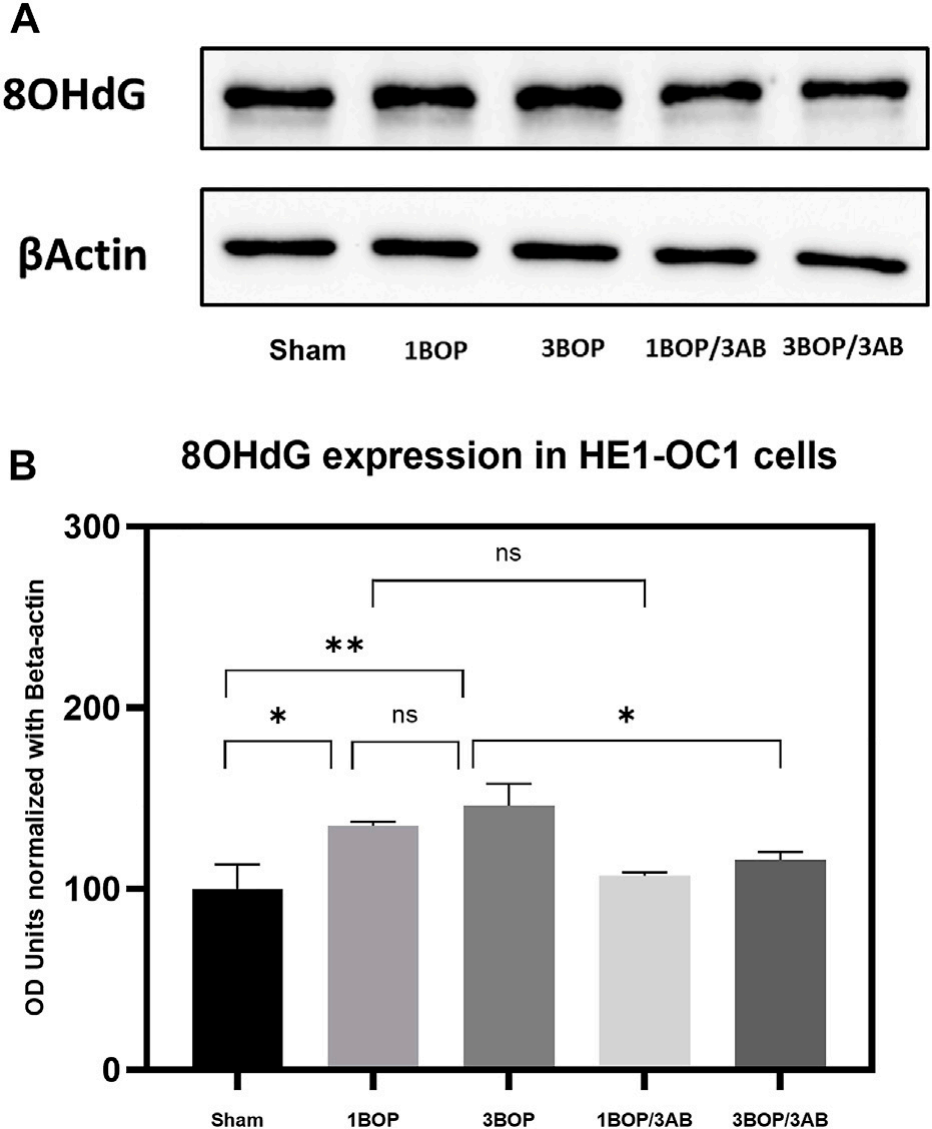
Assessment of 8-OHdG protein expressions by immunoblotting analysis in HEI-OC1 after BOP exposure. **(A)** The immunoblots shows the protein expressions of 8- OHdG levels in HEI-OC1 cells. **(B)** The histogram shows the semi-quantitative protein expression of 8-OHdG levels in HEI-0C1 cells normalized against the internal control (3-Actin. Statistical difference between the cell groups were (*p < 0.05; **p < 0.005) represented as Mean ± SEM (*n* = 3).

The changes in protein levels in BOP-exposed and 3AB-treated HEI-OC1 cells were assessed through immunoblot analysis. The immunoblot results showed a significant increase (F (4, 10) = 7.858) in PARP (Figure 6) protein level in 1-BOP (*p < 0.05) and 3-BOP cells (*p < 0.05) when compared with sham cells. But PARP level was not significantly different between 1-BOP and 3-BOP groups. Further, the PARP expression levels were significantly reduced (F (4, 10) = 7.858) in 1-BOP/3AB (*p < 0.05) and 3-BOP/3AB cells (*p < 0.05). Like PARP expression, the PAR (*p < 0.05 & ***p < 0.001; F (4, 10) = 12.29), 4HNE (**p < 0.005 & *p < 0.05; F (4, 10) = 12.51) and 8-OHdG (*p < 0.05 & **p < 0.005; F (4, 10) = 10.86) expression levels were significantly increased in 1-BOP and 3-BOP groups when compared with sham-exposed cells. In 1-BOP/3AB cells, the 4HNE expression was significantly lower against 1-BOP cells (*p < 0.05; F (4, 10) = 12.51), whereas the PAR (F and 8-OHdG does not exhibit any significant change. However, these PAR (4, 10) = 12.29) (Figure 7), 4HNE (F (4, 10) = 12.51) (Figure 8) and 8-OHdG (F (4, 10) = 10.86) (Figure 9) expression were significantly reduced in 3-BOP/3AB treated cell (*p < 0.05) when compared to 3-BOP group cells.

## Discussion

Blast-induced cochlear hair cell death was reviewed (Hamernik et al., 1984; Roberto et al., 1989) and reported in animal models such as mice (Cho et al., 2013), rats (Newman et al., 2015), and guinea pigs (Yokoi and Yanagita, 1984; Chen et al., 2013). Blast-induced cochlear synaptopathy has recently been reported in chinchillas (Hickman et al., 2018), and in rats (Niwa et al., 2016). Although morphological assessments such as cytochochleograms have reported the loss of hair cells with decreased spiral ganglion neurons and disrupted stereocilia, the molecular cascade of cell death following blast exposure remains underexplored, and such information will likely provide more knowledge on molecular targets for pharmacologic interventions.

Recently, *in vitro* studies on cochlear explants and hair cell lines demonstrated PARP over-activation leading to ototoxicity. Direct inhibition of PARP is reported in cochlear stria marginal cells by PARP1 knockdown which prevents ROS accumulation and PAR polymers, reduced mitochondrial membrane potential, and nuclear translocation of apoptosis-inducing factor (AIF) (Zhang et al., 2016; Jiang et al., 2018). In animal models, PARP overactivation in cochlear tissues (Shi and Nuttall, 2007) was reported to be inhibited by NAD^+^ supplementation in mice (Kim et al., 2016). PARP1 is shown to contribute to intense-sound-induced cochlear lateral wall damage by triggering inflammatory effects such as upregulation of intercellular adhesion molecule-1 (ICAM1) and P-selectin (Shi and Nuttall, 2007). Further, Tabuchi et al. (2003) reported acoustic injury-induced PARP over-activation in cochlea caused by 10 min exposure to a 2 kHz tone, 120 dB SPL in guinea pigs. Direct inhibition of PARP1 by PARP inhibitors boosts the anti-oxidative defense system by glutathione synthesis and interferes with the cell death pathway. Song et al. (2016) reported aminoglycoside-induced PARP over-activation in the cochlea and treatment with 3AB attenuated the PARP1-induced AIF translocation from mitochondria to nucleus thereby preventing cochlear cell death. Recently, cisplatin-induced ototoxicity was found to be attenuated in PARP1 deficient cochlear explants as well in wild-type explants treated with PARP1 inhibitor Pirenzipine (although not 3AB) (Tropitzsch et al., 2019). Intravenous 3AB is reported to reduce compound action potential shifts caused by 15/30 min of ischemic reperfusion injury in guinea pigs (Tabuchi et al., 2001). In addition to ototoxicity and cochlear ischemia, Murashita et al. (2006) reported that 3AB (500, 300, and 100 mg/kg intraperitoneal) is neuroprotective in an acoustic injury caused by 4 h exposure to 4 kHz tone of 110–128 dB SPL in mice. This evidence and our previous study of blast-induced cell death and neurotransmitter disruption led to our current study of investigating the effect of PARP inhibition on the *in vitro* model of blast-induced auditory hair cell death.

Being inspired by the other *in vitro* models which used high frequency, we used the model that delivers the low-frequency energy spectrum (Several kHz below 10 kHz), as most of the human hearing of speech falls in low frequency. In the present study of single and repetitive blast overpressure (impulse noise), we found that treatment with 3AB (3 mM for 24 h) significantly supports the cell viability and proliferation of HEI-OC1 cells following single BOP rather than repetitive BOP insults. Drug dose was determined by several previous biochemical assays and our doseresponse analysis. It has been demonstrated that the 50 μM of 3-aminobenzamide inhibits the Poly ADP-Ribose synthetase enzyme activity by 90% and incorporated only 0.23 nmol of NAD + per min per mg of protein (Purnell and Whish, 1980). It is well established that BOP insults result in free radicals in auditory hair cells (Du et al., 2013) especially since the 4HNE was found to be increased in both single/repetitive BOPs. The 4HNE is a highly reactive cytotoxic product formed by the peroxidation of membrane lipids which reflects the oxidative stress in tissues (Ayala et al., 2014). High levels of 4HNE can react with proteins and/or DNA adducts resulting multitude of cellular and molecular pathology (Eckl, 2003). These increased reactive oxygen species directly and/or indirectly through increased 4HNE lead to oxidative base damage and single-strand breaks of the nuclear and mitochondrial DNA (Lan et al., 2004). An increased single nucleotide base lesions of DNA of both sources were observed in BOP insults as reflected by the increased 8-hydroxy-2′-deoxyguanosine (8-OHdG). 8-OHdG is a potential marker of free-radical induced oxidative damage (Cadet et al., 2012) resulting in the over-activation of the PARP1. 3AB being a known potent inhibitor of PARP1, we confirmed the over-activation of PARP1 in both single and repetitive BOP insults through increased protein expression of PARP1 enzyme and its consequent decreased expression by 3AB treatment. Consequently, the PARylation (various length of PAR polymers–ADP Ribose units) was found to be increased in both single and repetitive insults. However, the PARylation is even higher in repetitive BOPs as expected.

The high PARP activity, PARylation, and increased oxidative stress lead to depolarization of mitochondrial membrane potential which paves way for the release of AIF or CytC from mitochondria leading to cell death (Baker and Staecker, 2012). Our results indicate that PARP inhibition by 3AB reduced the mitochondrial depolarization in both single and repetitive BOPs. Nrf2, a transcription factor that regulates the expression of antioxidant genes helps to maintain the integrity of the mitochondrial membrane potential (Kasai et al., 2020). A decreased Nrf2 leads to mitochondrial depolarization (Holmström et al., 2013) which is corroborated by our finding of reduced Nrf2 expression post-BOP and the same was found to be increased with 3AB treatment. Further, the Nrf2 regulates the recycling of the master redox regulator glutathione which helps to attenuate the BOP-induced oxidative stress (Harvey et al., 2009). The glutathione availability in the cytoplasm is determined by the expression of the glutathione-cysteine ligase-catalytic (GCLC) subunit which is a rate-limiting step in glutathione synthesis (Chen et al., 2005). The 3AB-treated HEI-OC1 cells showed a significant increase in the GCLC expression thereby supporting the cell viability by increasing the availability of the GSH. Further, the activity of the enzyme depends on ATP availability. Hence, we observed the ATP pool from both mitochondrial and glycolytic sources following BOP insults and with 3AB treatment. While the notion of PARP-dependent energy disruption is based on both ATP and NAD^+^ depletion, evidence indicates that NAD-independent ATP depletion could occur through the glycolytic pathway (Andrabi et al., 2014; Fouquerel et al., 2014). In addition to ADP ribosyl transferases like PARP, NAD^+^ serves as a substrate for other enzymes like Sirtuins and ADP-ribosylcylcases (Langelier et al., 2018; Strømland et al., 2019). Hence, NAD^+^ depletion by PARP1 over-activation would affect cellular energetics by other parallel pathways which warrants focused investigations. Evidence from other studies indicate that 3AB increases NAD^+^ pool and Sirtuin activity in cells (Thies and Autor, 1991; Gueguen et al., 2014). In alignment with other *invitro* studies, in both single/repetitive BOP insults, our findings indicate that the inhibition of PARP by 3AB revives the ATP pool from both mitochondrial and glycolytic sources at least following 1BOP.

Overall, given the limited therapeutic options for blast-induced hearing loss and tinnitus, our results on the effect of 3AB on mouse auditory hair cells indicate that inhibition of PARP1 might be a potential therapeutic approach for blast injury of the cochlea and auditory pathway. Hence further studies using 3AB or PARP inhibition of animal models of blast injury with consecutive pharmacokinetics and pharmacodynamics evaluation will be a promising path toward pre-clinical validation.

## Data Availability

The original contributions presented in the study are included in the article/supplementary material, further inquiries can be directed to the corresponding author.
